# The novel immunoglobulin super family receptor SLAMF9 identified in TAM of murine and human melanoma influences pro-inflammatory cytokine secretion and migration

**DOI:** 10.1038/s41419-018-1011-1

**Published:** 2018-09-19

**Authors:** Claudia Dollt, Julia Michel, Loreen Kloss, Susanne Melchers, Kai Schledzewski, Kathrin Becker, Andrea Sauer, Andreas Krewer, Franziska Koll, Astrid Schmieder

**Affiliations:** 1 Department of Dermatology, Venereology and Allergology, University Medical Center and Medical Faculty Mannheim, University of Heidelberg, and Center of Excellence in Dermatology, Theodor-Kutzer Ufer 1-3, 68167 Mannheim, Germany; 2 Institute for Transfusion Medicine and Immunology, Medical Faculty Mannheim, University of Heidelberg, Ludolf-Krehl-Str. 13-17, 68167 Mannheim, Germany

## Abstract

Melanoma is a highly immunogenic tumor with a good response to treatment with immune checkpoint inhibitors. Tumor-associated macrophages (TAMs) play an important immunosuppressive role in such tumors and have therefore been identified as possible future therapeutic targets in oncology. The aim of this study was to identify novel immunoregulatory receptors specifically expressed on TAM. Expression of Slamf9, a member of the signaling lymphocytic-activating molecule (Slam) immunoreceptor family, was found to be upregulated in a gene expression analysis of murine bone marrow-derived macrophages (BMDM) stimulated with tumor-conditioned medium of B16F1 melanoma cells. SLAMF9^+^ macrophages were identified in human and murine melanomas by using self-generated antibodies against human and murine SLAMF9. A comprehensive immunohistochemical analysis of tissue microarrays detected SLAMF9^+^ TAM in 73.3% of human melanomas, but also in 95.5% of naevi of melanoma patients and in 50% of naevi from healthy controls. In addition, 20% of melanomas and 2.3% of naevi from melanoma patients displayed a positive SLAMF9 expression also in melanocytic cells. No SLAMF9 expression was detected in naevus cells of healthy donors. Although SLAMF9 has no intracellular signaling motif, a comprehensive functional analysis revealed that the molecule was able to significantly enhance TNF-α secretion after LPS-stimulation. In addition, SLAMF9 delayed the wound closure of RAW 264.7 cells in a scratch assay, while proliferation and cell death were not affected. Taken together, SLAMF9 is a novel type-I-transmembrane receptor with immunomodulatory properties in macrophages. Further studies are required to evaluate whether SLAMF9 classifies as a promising future therapeutic target in melanoma.

## Introduction

Tumor-associated macrophages (TAM) play a crucial role in the development and progression of malignancies. Consequently, a high infiltration of TAM correlates with poor patient outcome in different tumor entities, such as mammary carcinoma^1^, lymphoma^2^, and malignant melanoma^3^. In general, macrophages are highly plastic phagocytic cells able to adapt to different environments. This plasticity is required since these cells play an important role in tissue homeostasis and host defense^4^. A simplified model of classification divides macrophages in pro-inflammatory M1-like macrophages and anti-inflammatory M2-like macrophages. During tumor initiation, TAM in most cases display a M1-like phenotype which eventually switches to a more M2-like-phenotype during tumor progression. This process leads to a mixed phenotype and a heterogeneous population of macrophages within the tumor which fulfill different functions^5^. By secreting various chemokines, TAM recruit regulatory T-cells (Tregs), Th2-cells and myeloid-derived suppressor cells (MDSCs) to the tumor site resulting in an immunosuppressive tumor microenvironment^6^. Moreover, TAM promote angiogenesis, tissue invasion of tumor cells and the formation of distant metastasis via the secretion of growth factors and matrix metalloproteinases^7^. Those properties qualify TAM as promising therapeutic targets.

In the past, our group has put effort into the characterization of TAM in malignant melanoma. This work resulted in the identification of Stabilin-1, Lyve-1, and Ms4a8a as potential new therapeutic targets in oncology^8–11^. In this study, we focused on the yet poorly characterized immunomodulatory Slamf9 surface receptor, which we identified on TAM, but also on a subset of malignant melanoma cells.

Proteins belonging to the family of signaling lymphocytic activation molecules (Slam) are immunomodulatory and cell-adhesive receptors^12^. They are expressed on the surface of a variety of hematopoietic cells, including T-cells^13^, NK-T-cells^14^, dendritic cells^15^ and macrophages^16^. This type of receptors are typically involved in self-ligand interactions and in most cases ligand binding leads to phosphorylation of immunoreceptor tyrosine-based switch motif (ITSM), a docking site for signaling adaptors such as SLAM-associated protein (SAP) and EWS-activated transcript 2 (EAT-2)^17^. In contrast to other Slam-family members, SLAMF9 lacks ITSM on its cytoplasmatic side and no possible signaling adaptors, signaling pathways activated by SLAMF9 and functions have yet been described^18,19^. Here we could show that SLAMF9 expressed on TAM is able to modulate the TNF-α-response of macrophages to LPS and affects cell migration and adhesion. These results provide evidence that SLAMF9 is functionally active despite lacking an intracellular phosphorylation side and is therefore worth analyzing in more detail.

## Results

### Slamf9 expression is upregulated by B16F1-derived tumor-conditioned medium in murine bone marrow-derived macrophages

**Fig. 1:**
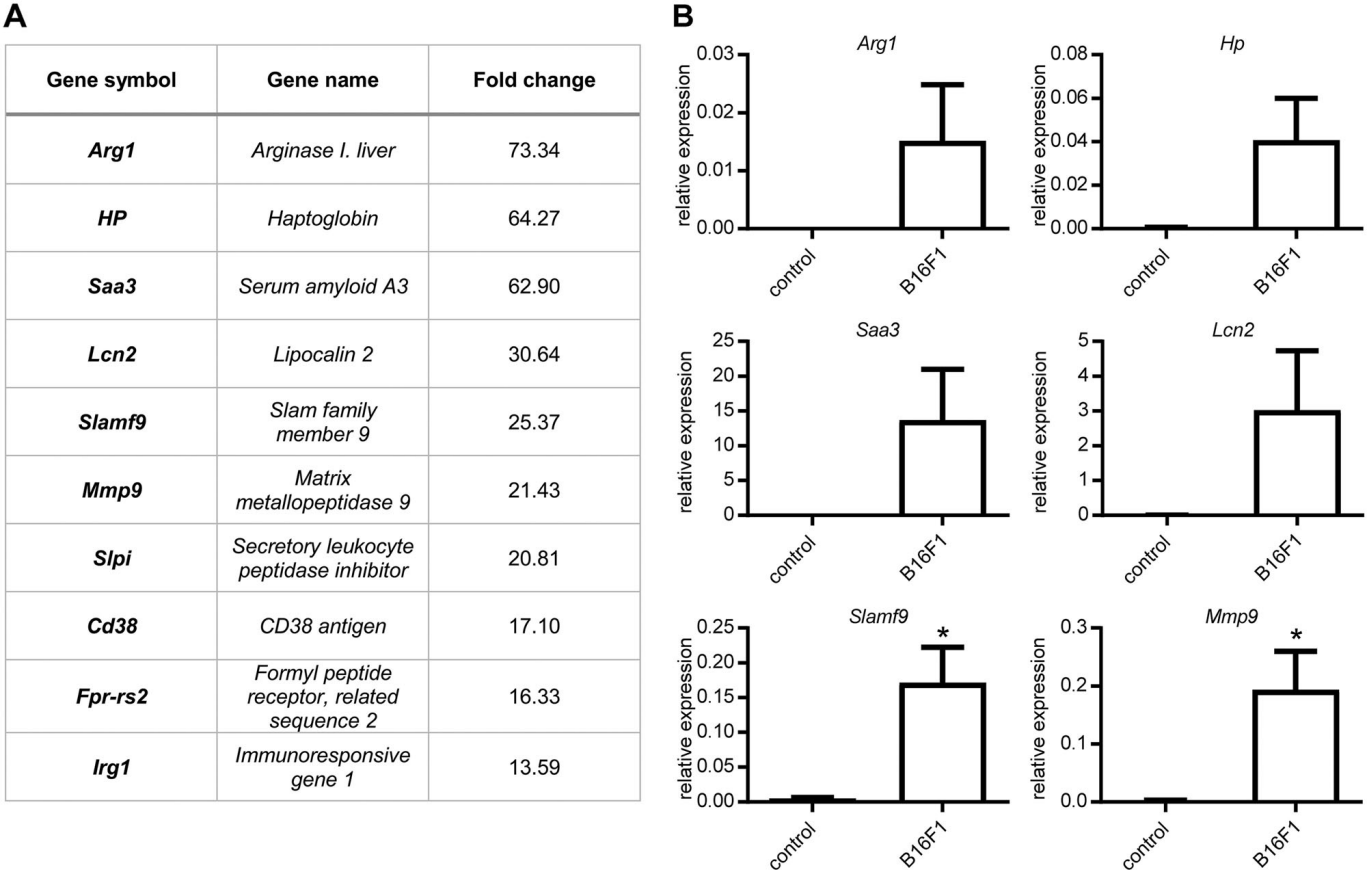
Gene expression profiling of TCM-induced TAM-like BMDM. **a** Microarray analysis of BMDM stimulated with B16F1 tumor-conditioned medium (TCM) in comparison to BMDM cultured in DMEM medium (control) (*n* = 3). To identify genes that were specifically upregulated by factors derived from B16F1 the fold change over control was calculated and an excerpt of the most highly regulated genes is illustrated in the table. **b** Validation of microarray data by qRT-PCR of BMDM stimulated with DMEM medium (control) or TCM of B16F1 cells. Values are normalized to an internal ß-actin control (*n* = 3). Data are presented as mean ± SEM

By cDNA-microarray analysis we examined the effect of tumor-conditioned medium (TCM) from B16F1 cells on the gene expression profile of bone marrow-derived macrophages (BMDM) in vitro in comparison to BMDM treated with culture medium as control. In total, 567 genes showed a significant upregulation while 861 genes were significantly downregulated. The ten most highly upregulated genes in the TCM-treated group are shown in Fig. 1a. Of these genes, six were validated by qRT-PCR (Fig. 1b) showing significantly enhanced gene expression of Slamf9 and Mmp9 (Fig. 1b).

### CD11b^+^ TAM of subcutaneously grown B16F1 tumors express Slamf9

**Fig. 2:**
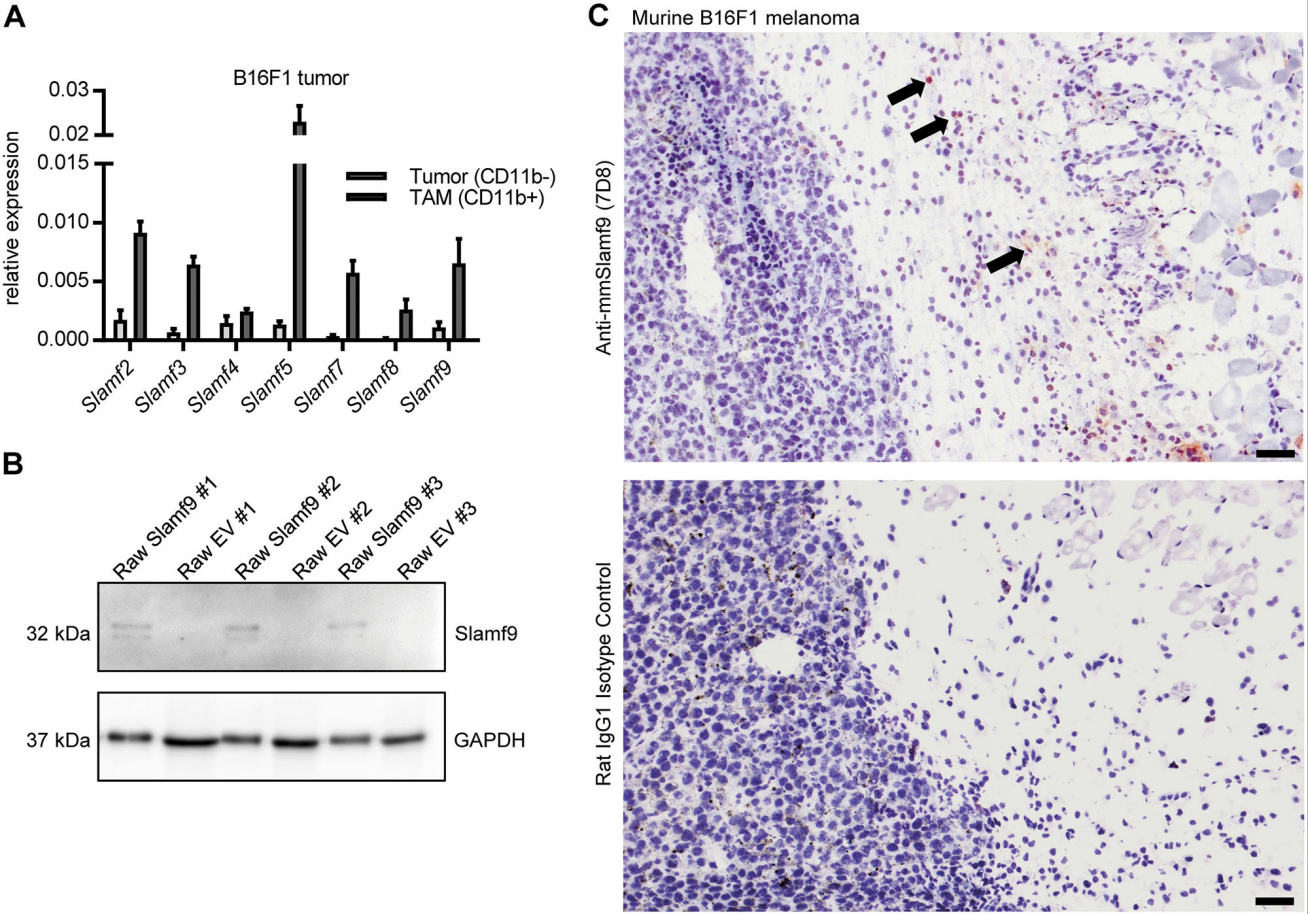
Slamf9 is expressed in murine melanoma-derived TAM. **a** 1 × 10^6^ B16F1 cells were injected subcutaneously into the flank of C57BL/6 wild-type mice. The tumors were excised 14 days after the injection. Tumors were homogenized and cells were separated according to CD11b expression by magnetic cell sorting. The expression of Slamf members was assessed by qRT-PCR analysis. Values are normalized to an internal ß-actin control (*n* = 3). Data are presented as mean ± SEM. **b** Western blot analysis of three independent clones of transgenic Slamf9^+^ RAW 264.7 cells and EV RAW 264.7 cells using the self-generated monoclonal anti-mmSlamf9 antibody, rat IgG1 isotype control or anti-GAPDH antibody. **c** Immunohistological staining of acetone-fixed cryo-sections of murine B16F1 tumors using our self-generated monoclonal anti-mmSlamf9 antibody or rat IgG1 isotype control. One representative picture is shown, scale bar = 100 µm

Proteins of the Slam family are expressed by hematopoietic cells; however, their expression in TAM has yet not been under investigation. Expression analysis of the Slam-genes was conducted in CD11b^+^ TAM isolated from subcutaneously grown B16F1 tumors and compared to expression levels in the CD11b^−^ fraction of the isolated cells. While expression of Slamf1 and Slamf6 could not be detected in TAM, Slamf9 and all other members were found to be expressed at higher levels in the CD11b^+^ fraction (Fig. 2a). To evaluate the presence of Slamf9 on protein level, a rat monoclonal antibody against mmSlamf9 was generated and its specificity confirmed by immunocytochemical (ICC) staining of transgenic Slamf9^+^ RAW 264.7 cells (Supplemental Fig. 1B). In addition, Western-blot analysis of these transgenic cells was performed, which identified a specific double band at ~32 kDa corresponding to the glycosylated estimated Slamf9 protein length of 285 amino acids (aa) (Fig. 2b). This antibody was used to detect Slamf9^+^ macrophages in subcutaneously grown murine B16F1 melanomas. Immunohistochemical staining revealed an accumulation of Slamf9^+^ macrophages predominantly in the periphery of this tumor (Fig. 2c).

### Murine Slamf4, -7, -8, and -9 are induced by IFN-γ in BMDM

**Fig. 3:**
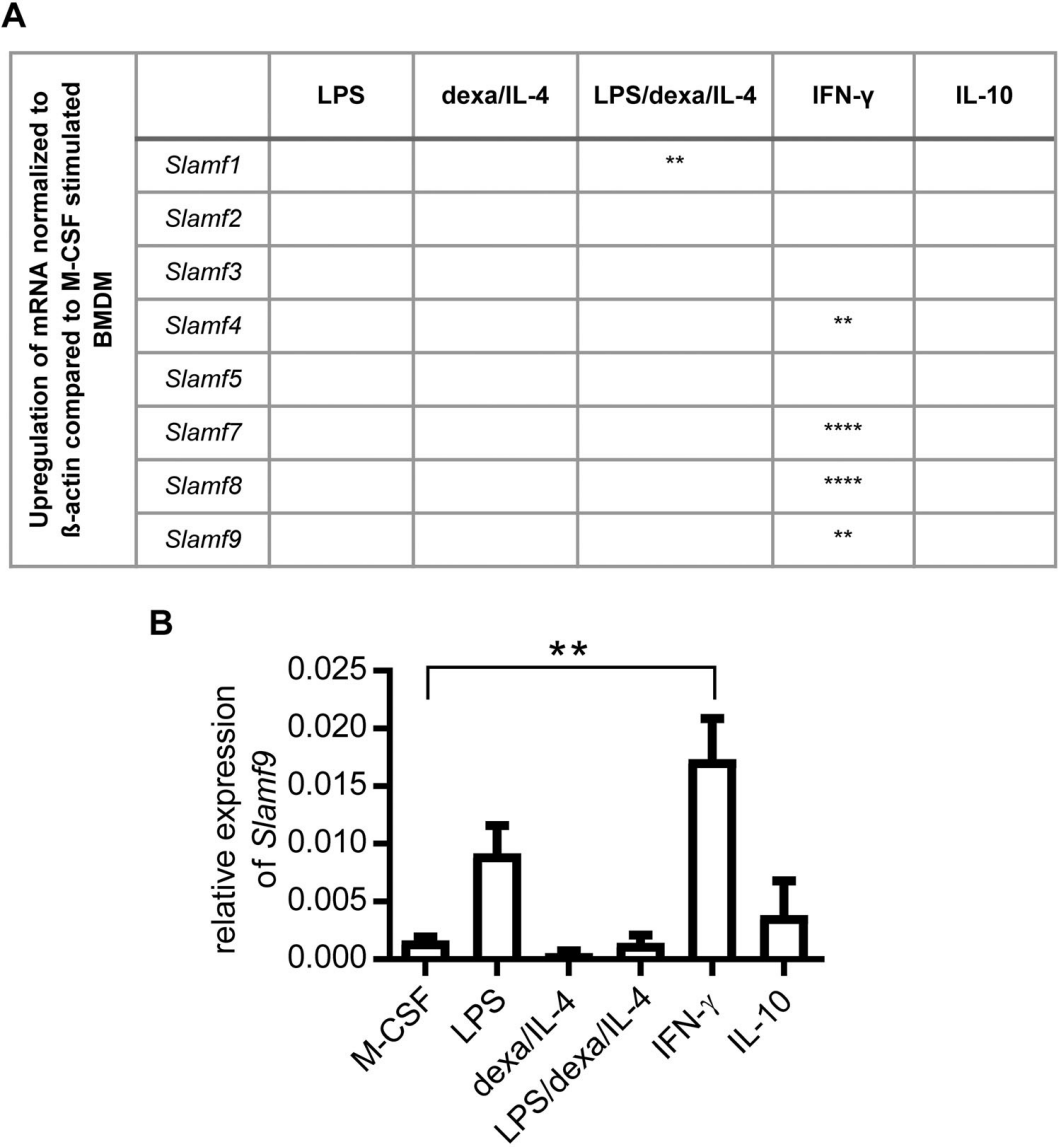
Slamf-9 is induced in BMDM by stimulation with IFN-γ. After 4 days of stimulation with M-CSF, medium of BMDM was exchanged by new M-CSF containing culture medium supplemented with different combinations of pro- and anti-inflammatory mediators as indicated for three further days. The gene expression of Slamf members was examined by qRT-PCR. Normalization was performed based on an internal ß-actin control. **a** Summary of significant mRNA upregulation of Slamf members stimulated as indicated and compared to M-CSF stimulated BMDM (*n* = 3). **b** Bar chart of the relative gene expression of Slamf9 mRNA (normalized to ß-actin) assessed by qRT-PCR (*n* = 3). Data are presented as mean ± SEM

In the tumor microenvironment, a broad mixture of pro- and anti-inflammatory factors influences the polarization of macrophages^20^. To identify possible mediators, which could be important for the induction of Slam proteins, MCSF-differentiated BMDM were stimulated for 4 days with different combinations of pro- and anti-inflammatory agents. Expression of Slamf-2, -3, -5, and -6 could not be evoked by the tested stimulatory combinations. However, Slamf1 was upregulated by the combined stimulation with LPS/dexa/IL-4, and IFN-γ stimulation led to a significant increase of Slamf4, -7, -8, and -9 mRNA (Fig. 3a). Slamf9 expression was also increased by treatment of BMDM with LPS, even though not significantly (Fig. 3b). Taken together, Slamf9 and other family members are predominantly induced by pro-inflammatory mediators.

### Human Slamf9 is induced by WM115-derived supernatant and M-CSF/LPS in peripheral blood monocytes (pBMs)

**Fig. 4:**
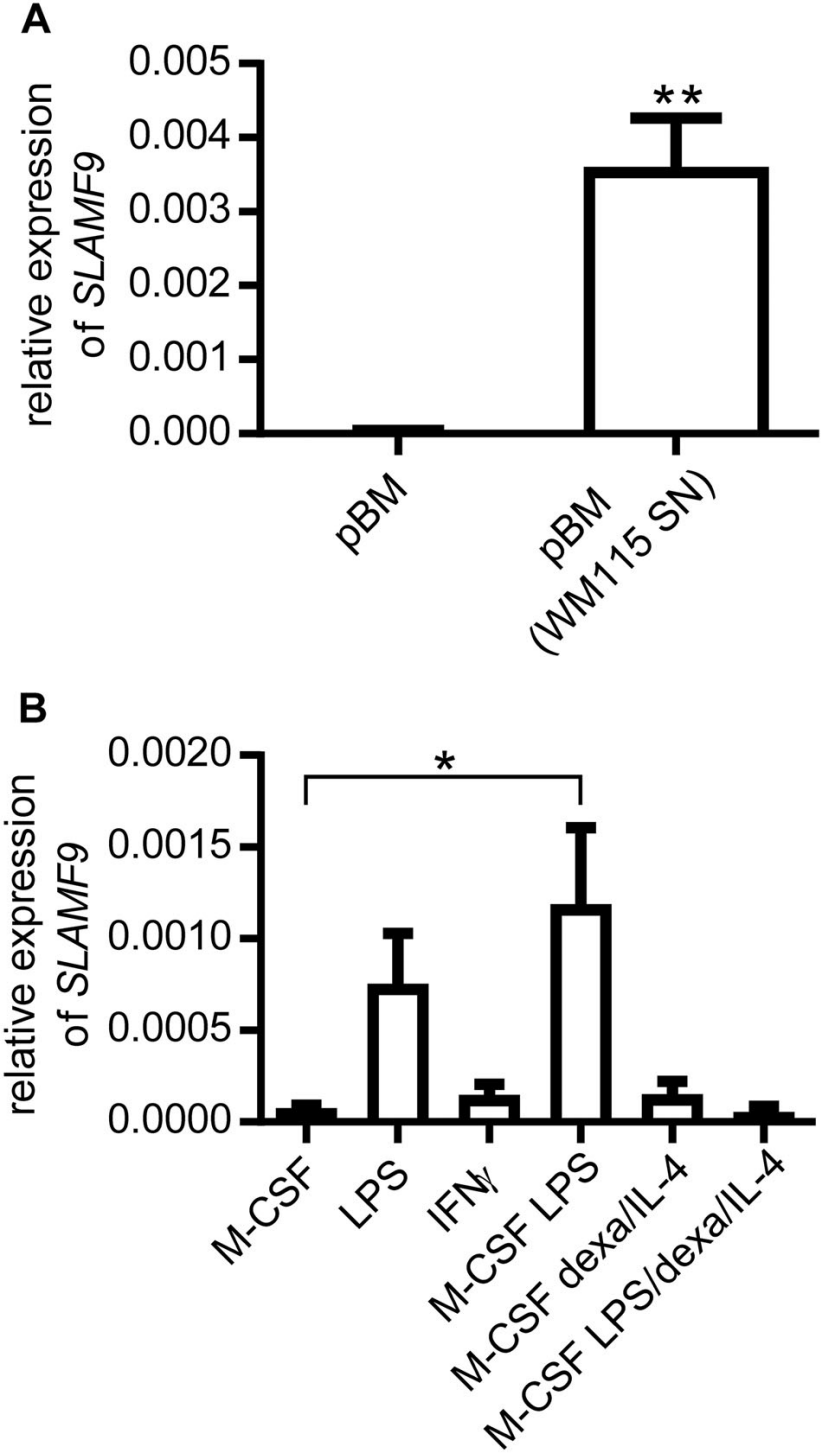
SLAMF-9 is induced in human pBM by TCM or LPS. **a** CD14^+^ pBM were isolated from healthy donor-derived buffy coat samples. pBM were stimulated for 7 days either with control medium or TCM from WM115 human melanoma cells. Expression of human Slamf9 was evaluated by qRT-PCR analysis (*n* = 4). Data are presented as mean ± SEM. **b** pBM were stimulated for 7 days with different combinations of pro- and anti-inflammatory mediators as indicated. The expression level of Slamf9 was determined by qRT-PCR analysis. In both cases the values were normalized to an internal ß-actin control (*n* = 3). Data are presented as the mean ± SEM

It is well known that species-specific differences in the marker profile of TAM exist. Hence, previous findings were transferred to a human setting. CD14^+^ monocytes were isolated from peripheral blood buffy coats and cultured in TCM derived from the human melanoma cell line WM115. The melanoma-derived supernatant significantly induced gene expression of human Slamf9 (Fig. 4a). In addition, mirroring the previous mouse experiment, pBMs were stimulated with different mixtures of pro- and anti-inflammatory agents. Interestingly, treatment of pBMs with IFN-γ had no effect on human Slamf9 expression; neither did exposure to combinations containing IL-4 and dexamethasone. Only M-CSF/LPS achieved a significant induction of Slamf9 mRNA (Fig. 4b).

### 73.3% of all human melanocytic lesions are infiltrated by SLAMF9^+^ macrophages

**Fig. 5:**
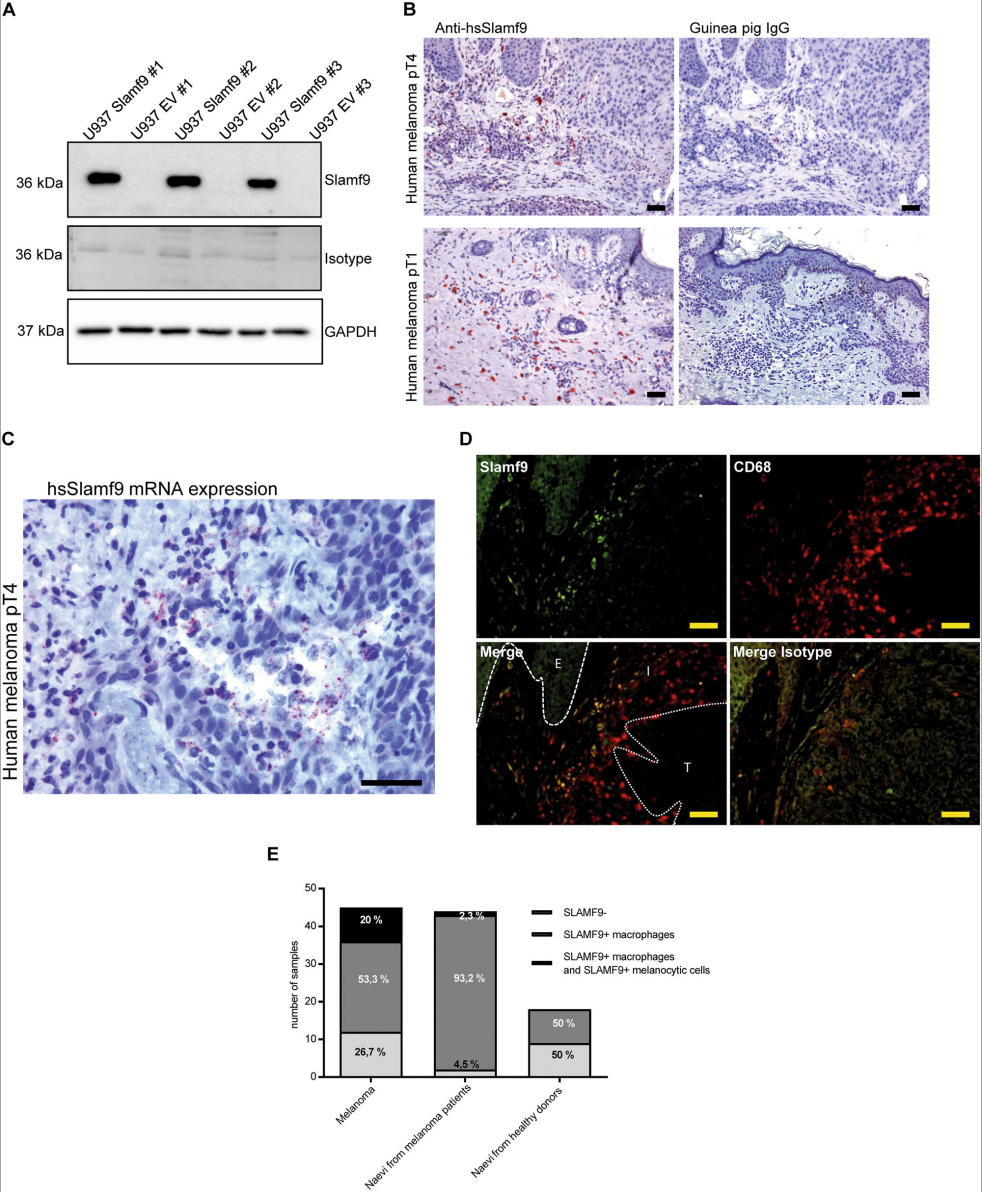
Identification and quantification of SLAMF9^+^ cells in benign and malignant melanocytic lesions. **a** Western blot analysis of three independent transgenic SLAMF9^+^ U937clones and EV U937 clones was performed to validate specificity of our self-generated polyclonal anti-hsSLAMF9 antibody. The respective guinea pig IgG was used as isotype control and GAPDH was used as a loading control. **b** Human melanoma specimens were immunohistochemically stained with the self-generated polyclonal anti-hsSLAMF9 antibody and guinea pig IgG isotype control. Representative pictures of pT1 and pT4 melanoma specimens are shown, scale bar = 100 µm. **c** In situ hybridization was performed to visualize Slamf9 mRNA in formalin-fixed paraffin-embedded melanomas. One representative picture of a pT4 melanoma specimen is shown, scale bar = 100 µm. **d** Immunofluorescent double staining of SLAMF9 (green) and CD68 (red) as well as the respective guinea pig IgG and mouse IgG isotype controls of a human pT4 melanoma was performed, scale bar = 100 µm. E Epidermis, I inflammatory infiltrate, T Tumor. **e** Quantification of SLAMF9^+^ cells in tissue microarrays with benign and malignant melanocytic lesions. Two core sections of each specimen were evaluated after immunohistochemical staining of SLAMF9. The percentage of samples containing SLAMF9^+^ TAM only or SLAMF9^+^ TAM plus SLAMF9^+^ tumor cells was histopathologically assessed

A polyclonal antibody against hsSLAMF9 was generated. The specificity of the antibody was tested by ICC staining of transgenic SLAMF9^+^ U937 cell lines (Supplemental Fig. 1A). In addition, Western blot analysis of these cells was performed identifying a specific band at about 36 kDa corresponding to the glycosylated estimated hsSLAMF9 protein length of 289 aa (Fig. 5a).

This antibody was used to detect SLAMF9 in human melanocytic lesions. First, one exemplary slide of each pathological melanoma stage (stages I–IV) was immunohistochemically stained, revealing the presence of SLAMF9^+^ inflammatory cells in all four stages (Fig. 5b, Supplementary Fig. 2). In situ hybridization confirmed the existence of inflammatory cells positive for Slamf9 mRNA in human melanomas and further identified some Slamf9-mRNA^+^ melanoma cells (Fig. 5c). Finally, immunofluorescent double staining of SLAMF9 and CD68 showed, that most of the SLAMF9^+^ inflammatory cells are TAM (Fig. 5d). A comprehensive immunohistochemical analysis of two tissue microarrays (TMA) composed of 107 melanocytic lesions (45 malignant melanomas, 44 naevi of melanoma patients and 18 naevi of healthy controls) detected SLAMF9^+^ cells in 79.5% of all analyzed tissue samples. SLAMF9^+^ TAM were spotted in 73.3% of melanomas and in 95.5% of naevi from melanoma patients, while only 50% of naevi from healthy donors were infiltrated by SLAMF9^+^ macrophages. As already seen by in situ hybridization, SLAMF9 expression in melanoma and naevi of melanoma patients was not limited to macrophages. Twenty percent of the examined melanomas and 2.3% of the naevi of melanoma patients also showed SLAMF9 expression by melanocytic cells. However, no SLAMF9^+^ neavus cells were detected in naevi of healthy controls (Fig. 5e).

### In macrophages SLAMF9 enhances TNF-α secretion after LPS stimulation and reduces their migratory capacity

**Fig. 6:**
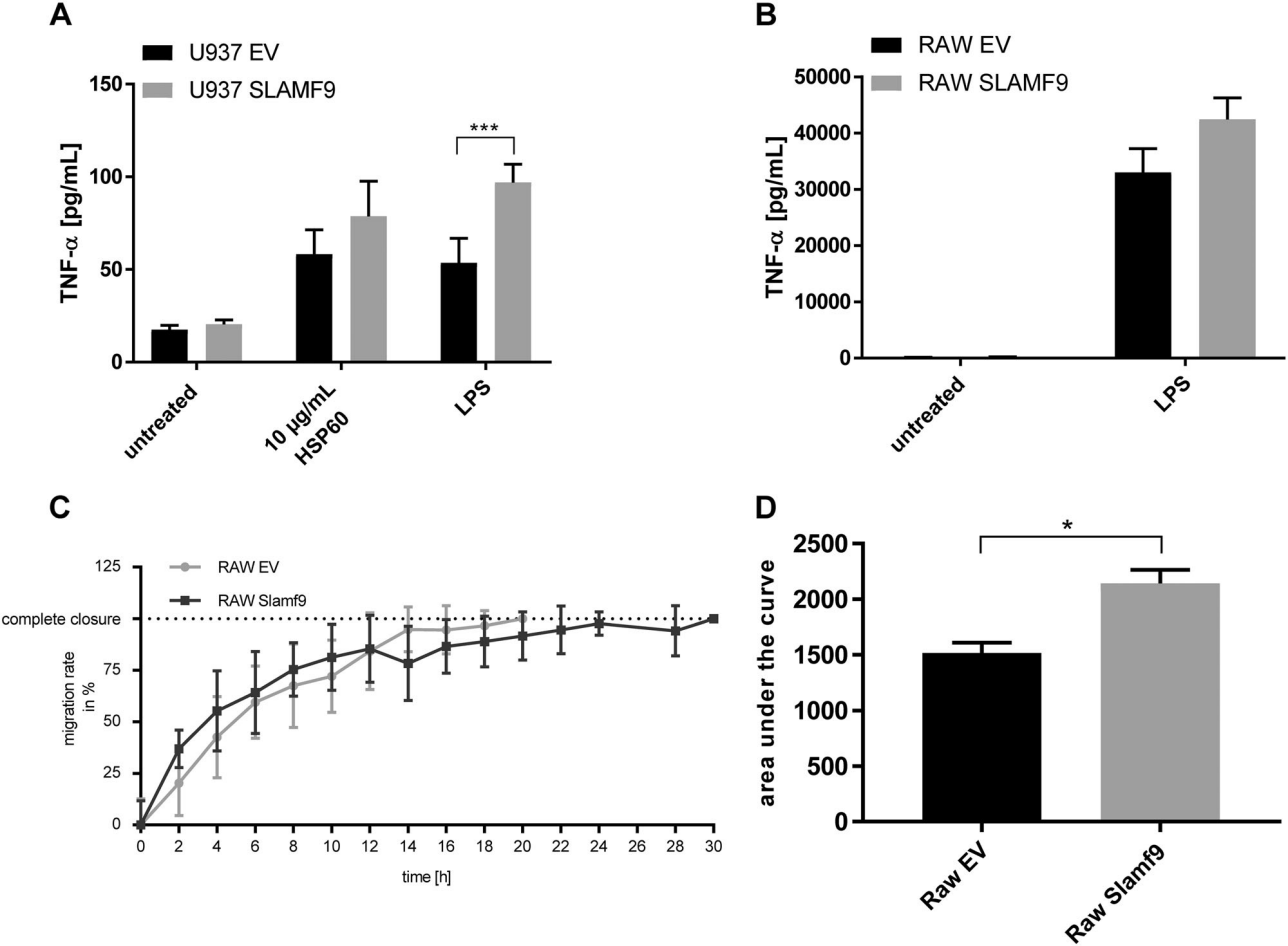
In macrophages SLAMF9 influences TNF-α secretion upon pro-inflammatory stimulation and reduces cell migration. **a** Transgenic SLAMF9^+^ U937 cells and EV U937 cells were stimulated for 48 h with 10 ng/ml PMA prior to treatment with 1 µg/ml LPS or 10 µg/mL HSP60 for 6 h. TNF-α concentration in the cell culture supernatant was determined by ELISA (*n* = 3), data are presented as mean ± SEM. **b** Transgenic Slamf9^+^ RAW 264.7 cells and EV RAW 264.7 cells were stimulated with 1 µg/ml LPS for 6 h. TNF-α concentration in the cell culture supernatant was determined by ELISA (*n* = 3), data are presented as mean ± SEM. **c** An artificial scratch was caused to 100%-confluent monolayers of transgenic Slamf9^+^ RAW 264.7 cells and EV RAW 264.7 cells. The width of the gap was documented every two hours for a total of 30 h. One out of three experiments is depicted. **d** Area under the curve of three independent scratch assays performed with transgenic Slamf9^+^ RAW 264.7 cells and EV RAW 264.7 cells was calculated

Next, we focused on the function of SLAMF9 in macrophages. Since it has been demonstrated that SLAMF5, the closest homolog of SLAMF9^21^, enhances TNF-α secretion in macrophages upon LPS stimulation^22^, we treated transgenic U937 cells with LPS for 6 h after differentiation to macrophages with phorbol 12-myristate 13-acetate (PMA). This LPS treatment upregulated both the mRNA expression (figure not shown) and protein secretion of TNF-α in U937-derived macrophages, remarkably to significantly greater extend in SLAMF9^+^ cells compared to U937 EV cells (Fig. 6a). Stimulation of the U937 cells with another TLR-agonist, namely HSP60, also induced an increased secretion of TNF-α in SLAMF9^+^ U937 cells, although without reaching statistical significance (Fig. 6a). To validate the result obtained with LPS in a murine macrophage-like cell line, transgenic RAW 264.7 cells were stimulated with LPS for 6 h and TNF-α secretion was analyzed by ELISA. Here, a tendency towards a higher TNF-α secretion in SLAMF9^+^ cells compared to RAW 264.7 EV cells was identified, but the difference was not significant.

In addition, the influence of SLAMF9 overexpression on basic cellular functions was examined. We focused on the impact of SLAMF9 on cell proliferation, cell death and migratory capacity. While no differences with regard to proliferation and H_2_O_2_-induced cell death were observed in Slamf9^+^ RAW 264.7 cells and SLAMF9^+^ U937 cells compared to EV controls (Supplemental Fig. 3A-D), SLAMF9 overexpression impaired the migration of RAW 264.7 cells in a scratch-based wound healing assay reaching statistical significance (Fig. 6c, d).

## Discussion

The Slam-family consists of nine transmembrane receptors, SLAMF1 to SLAMF9. Excluding SLAMF2 and SLAMF4, which interact with each other, they are homophilic receptors and ligand engagement leads to phosphorylation of ITSM^23^. However, not all Slamf members bear ITSM. SLAMF2 has a glycophosphatinositol-anchor instead of ITSM and SLAMF8 and SLAMF9 lack intracellular signaling motifs^23^.

The Slam-family is involved in a great variety of important immune and cell-biological functions. They regulate proliferation, cytotoxicity, and cytokine production of T-lymphocytes, modulate lytic activity, cytokine production, and MHC-independent cell inhibition of NK cells, B cell activation and memory generation, neutrophil and macrophage killing, and platelet aggregation^14,17^.

SLAMF9 is the most recently identified member of the Slam-family. Like nearly all other Slamf-receptors, it has an extracellular amino-terminal variable immunoglobuline-like domain, a membrane proximal constant immunoglobuline-like domain, a transmembrane region, but no known signaling motifs in its intracellular tail^24^. Slamf9 mRNA was detected in a variety of immune cells such as human monocytes, T cells, B cells, and DCs. So far, no signal transduction mode and no ligand have been identified^18,21,25^.

In this study, Slamf9 was discovered by gene expression analysis of BMDM stimulated with TCM from B16F1 melanoma cells. In humans, melanoma is a highly immunogenic tumor resistant to conventional chemotherapeutic approaches, but shows a good response to immune checkpoint inhibitors like pembrolizumab^26,27^. Because of the great immunological role of Slamf members, the focus was set on the expression and functional analysis of human and murine SLAMF9 in macrophages infiltrating both benign and malignant melanocytic lesions.

First, we analyzed the mRNA expression of other Slamf members since these proteins are known to work as self-ligands or to interact with each other^28^. The mRNA of nearly all Slamf members could be detected in ex vivo isolated TAM from murine B16F1 tumors with Slamf5 showing the highest expression. In general, SLAMF5 is expressed on B-cells, monocytes and platelets^24^. Binding of SLAMF5 to Slamf5-Ig and CD3 leads to an enhanced IFN-γ production in human lymphocytes^15^. In addition, it is an important molecule for autoimmune disease by maintaining germinal center B-cell tolerance^29^. Since SLAMF5 is the closest homolog to SLAMF9 a possible interaction between these related proteins cannot be excluded. This should be verified in the future, especially since it has been shown that SLAMF5 enhances the LPS-induced secretion of TNF-α and other pro-inflammatory mediators in murine macrophages^23^. We were able to show that human SLAMF9 has the same effect in transgenic human U937 macrophage-like cells although Slamf9 surface expression cannot be verified with our self-made antibodies.

The induction of human and murine SLAMF9 requires pro-inflammatory stimulators such as IFN-γ and LPS or TCM from melanoma cells. At first, this fact seems contradictory as TAM are immunosuppressive tumor stroma cells, but in fact IFN-γ plays a crucial role in tumor progression. In a subcutaneous ovarian cancer model, it has been demonstrated that the injection of IFN-γ into the tumor induced the expression of Programmed Death Ligand 1 (PD-L1) and subsequently promoted tumor growth^30^. Therefore, targeting SLAMF9 may be beneficial to enhance anti-tumor immunity.

Apart from its immunological functions, SLAMF9 inhibits the migratory behavior of macrophages, probably by increasing adhesion. This has also been shown for other Slamf members, such as SLAMF8. In Slamf8^−/−^ knockout mice the migration rate of macrophages was enhanced in a Nox2-dependent manner leading to enhanced reactive oxygen species (ROS) production^31,32^. In addition it has been shown that T cells expressing SLAMF1 exhibit increased clumping and that SLAMF5 mediates T-B-cell adhesion^33^. Since both SLAMF8 and SLAMF9 lack intracellular signaling motifs, SLAMF9 may also reduce migration by preventing the assembly of the Nox2 complex potentially both in TAM and tumor cells.

SLAMF9 plays an important role in melanoma biology, which is highlighted by the fact that we found SLAMF9^+^ TAM not only in subcutaneously implanted murine B16F1 melanoma but also in 73.3% of human melanomas stained with our self-generated peptide anti-SLAMF9 antibody. Strikingly, in 20% the melanoma cells themselves were SLAMF9 positive while only 2.3% of melanocytic naevus cells derived from melanoma patients and none of the naevi from healthy donors showed SLAMF9 expression. SLAMF9 expression in melanocytic lesions may therefore indicate a genetic predisposition to develop a malignant melanoma. With regard to macrophage infiltration, SLAMF9 was detected in a high percentage of benign and malignant melanocytic lesions and could therefore not be correlated with malignancy. Further studies are needed to evaluate the use of SLAMF9 as marker for melanoma.

Taken together, SLAMF9 is a receptor belonging to the immunoglobuline-receptor superfamily expressed in melanocytic lesions. In this study, we provide evidence for SLAMF9’s capacity to modulate the proinflammatory response and migratory behavior of macrophages despite lacking intracellular signaling motifs. Further studies are recommended to identify possible ligands and the distinct signaling pathways activated by SLAMF9. In addition, the exact functional impact of SLAMF9^+^ TAM for melanoma development and progression needs to be evaluated in more detail to identify its potential as a future therapeutic target.

## Materials and methods

### Human samples

The study was performed according to federal laws and regulations as well as institutional policies. We obtained ethical approval from the local ethical committee (reference number: 2010-318N-MA). Written informed consent was obtained from all patients and data were analyzed anonymously.

### Mice

C57BL/6 were purchased from The Jackson Laboratory. All mice were housed under specific pathogen-free conditions at the animal facility Mannheim. Animal experimental protocols were approved by the animal ethics committee (Regierungspräsidium Karlsruhe, Az: 35-9185.81/G-42/14)

### Cell lines

The human monocytic cell line U937 (CRL-1593.2™, ATCC®, Wesel, Germany) and the human melanoma cell line WM115 (CRL-1675™, ATCC®, Wesel, Germany) were cultured in RPMI-1640 (Gibco by Thermo Fisher Scientific, Darmstadt, Germany) supplemented with 10% fetal calf serum (FCS, Biochrom, Berlin, Germany), 100 U penicillin, as well as 100 mg/l streptomycin (Pen/Strep, Biochrom, Berlin, Germany). The murine macrophage-like RAW 264.7 cells (TIB-71™, ATCC®, Wesel, Germany), the murine melanoma cells B16F1 (CRL-6323™, ATCC®, Wesel, Germany) were cultivated in DMEM (Gibco by Thermo Fisher Scientific, Darmstadt, Germany) with 10% FCS, 1% Pen/Strep (DMEM complete). HEK 293T/17 (CRL-11268™, ATCC®, Wesel, Germany) cells were maintained in DMEM complete plus 100 mM sodium-pyruvate (Sigma-Aldrich, Munich, Germany). All cell lines were cultivated at 37 °C in an atmosphere enriched with 5% CO_2_.

### Generation of TCM

The respective tumor cell line was allowed to reach 50% confluency. Medium was removed and the monolayer was washed once with PBS (Gibco by Thermo Fisher Scientific, Darmstadt, Germany). New culture medium was added and incubated with the cells for 48 h at 37 °C and 5% CO_2_. Thereafter, the conditioned cell culture medium was collected and centrifuged to obtain cell-free supernatants.

### Tumor models

A total of 1 × 10^6^ B16F1 cells were injected subcutaneously into the flank of 8–10 weeks old female mice. After 14 days of tumor growth, mice were sacrificed and the excised tumors were further processed for CD11b^+^ cells isolation.

### Generation of BMDMs

Murine bone marrow cells were generated by flushing of femur and tibia as described previously^11^ and were seeded with a concentration of 1 × 10^6^ cell/ml DMEM complete supplemented with 30 ng/ml M-CSF (Peprotech, Hamburg, Germany) and incubated for 4 days at 37 °C and 7.5% CO_2_ to allow differentiation from monocytes to macrophages. Thereafter, medium was replaced by DMEM complete containing M-CSF plus different combinations of pro-and anti-inflammatory mediators, namely IL-4 (10 ng/ml, Peprotech, Hamburg, Germany), Dexamethasone (5 × 10^7^ M, Sigma-Aldrich, Munich, Germany), IFN-γ (10 ng/ml, Peprotech, Hamburg, Germany), IL-10 (10 ng/ml, Peprotech, Hamburg, Germany) and LPS (1 µg/ml, Invitrogen, by Thermo Fisher Scientific, Darmstadt, Germany) or TCM from B16F1 as indicated in the result section. The cells were incubated for three further days.

### Isolation of murine macrophages

Murine tumors were excised and rinsed in PBS before being homogenized by chopping. The homogenate was digested in 10 ml RPMI supplemented with collagenase IV (190 U/ml, Genaxxone, Ulm, Germany) and DNAse I (500 U/ml, Sigma-Aldrich, Munich, Germany) at 37 °C for at least one hour. A single cell suspension was generated by passing the lysate through a 100 µm cell strainer. The suspension was washed with PBS and CD11b^+^ cells were isolated by magnetic-activated cell sorting (MACS) using anti-CD11b MicroBeads (Miltenyi, Bergisch-Gladbach, Germany).

### Isolation of human peripheral blood monocytes

CD14^+^ cells were isolated from buffy coats from healthy donors obtained from Red Cross Blood Service, Baden-Württemberg. Peripheral blood mononuclear cells were separated from whole blood by gradient centrifugation and then monocytes were isolated by MACS using anti-CD14 MicroBeads (Miltenyi, Bergisch-Gladbach, Germany). For differentiation, 1 × 10^6^ cells/ml were seeded in X-VIVO™ 15 (Lonza, Cologne, Germany) and cultivated for 7 days at 37 °C and 7.5% CO_2_. pBM were stimulated with M-CSF (100 ng/ml, Peprotech, Hamburg, Germany), IL-4 (10 ng/ml, Peprotech, Hamburg, Germany), Dexamethasone (1 × 10^–7^ M; 1000 U/ml, Sigma-Aldrich, Munich, Germany), IFN-γ (10 ng/ml, Peprotech, Hamburg, Germany) and LPS (1 µg/ml, Invitrogen by Thermo Fisher Scientific, Darmstadt, Germany) or TCM from WM115 mixed 1:1 with fresh X-VIVO™ 15 as indicated.

### Generation of anti-murine and anti-human SLAMF9-antibodies

For the generation of a monoclonal antibody against murine SLAMF9, the N-terminal part of mmSlamf9 (IMAGE cDNA clone:4458767, amino acid 1 to 230) was cloned in fusion to murine Fc fragment in vector pCDN3.1. Stably transfected HEK293 cells secreting NT-mmSlamf9-Fc into cell culture supernatant allowed purification of recombinant soluble Slamf9 protein via Protein A gel columns (invivo Biotech, Hennigsdorf). For immunization, three rats were intraperitoneally injected with an immunization cocktail containing 100 µg purified NT-mmSlamf9-Fc in 100 μl PBS and 100 μl incomplete Freunds adjuvants. After three routine immunizations (4 weeks schedule) rats were sacrificed three days after the final immunization (200 µg NT-mmSlamf9-Fc in 200 μl PBS without Adjuvans) step. B cells were isolated form the spleen and fused with X63 Ag8.653 myeloma cells with Polyethylene glycol 4000 (Sigma, Taufkirchen) in order to obtain hybridoma cells. Cells were diluted in RPMI Glutamaxx (Gibco) supplemented with HAT (Sigma, Taufkirchen) to 10 cells/ml and transferred to 96-well plates. Growing hybridoma colonies were screened for binding full length mmSLAMF9 protein (crudes lysate of mmSlamf9 transgenic HEK293 cells) in a dot blot test. The clone 7D8 (rat isotype IgG1) was selected, expanded and specifity further confirmed by immunohistochemistry and western blot analysis.

A polyclonal antibody against human SLAMF9 was generated in guinea pig, targeting the peptide sequence RNRMKLRKEAKPGSSPA referring to amino acid 273 to 289 of the C-terminus (protein ID NP_254273.2). The synthetized peptide was coupled to KLH carrier protein and used as immunogen in a commercial process (PSL, Heidelberg). The polyclonal anti-hsSLAMF9 antibody was purified by affinity chromatography using a peptide-specific column provided by PSL, Heidelberg. The antibody was validated by immunohistochemical stainings of hsSLAMF9 transgenic HEK293 cells (data not shown) and Western-blot using recombinant expressed protein.

### Immunohistochemistry

Cryostat sections (7 µm) from murine melanoma tumors were air-dried and acetone-fixed. Paraffin-embedded human tissue samples were dewaxed as described before using a decreasing xylene/alcohol series and heat-induced antigen retrieval was performed. Subsequently, specimens were incubated with 0.3% peroxide, 2% BSA and the primary antibody. For mice, the rat anti-mmSLAMF9 monoclonal antibody and its isotype control (rat-IgG1, R&D, Wiesbaden, Germany) and for human tissue the guinea pig anti-hsSLAMF9 and its isotype control (guinea pig IgG, Novus Bio, Wiesbaden, Germany) were used. After incubation with the appropriate HRP-labeled secondary antibody (for murine Slamf9 anti-rat HRP kappa light chain, for human Slamf9 anti-guinea pig HRP; both Dianova, Hamburg, Germany), AEC chromogen solution (Biozol, Eching, Germany) was applied for visualization. Mayer’s Haemalaun (Merck, Darmstadt, Germany) was used for counterstaining. Pictures were taken with a Leica DCRE microscope, Leica DC500 camera, and software system (Leica, Wetzlar, Germany).

### Immunofluorescence

Specimens were fixed and prepared as described before. After washing, blocking was performed with 2% BSA in PBS. Primary antibody (self-generated guinea pig anti-human SLAMF9, mouse anti-human CD68 (Dako by Agilent, Waldbronn, Germany), mouse IgG and guinea pig IgG (Novus Bio, Wiesbaden, Germany), was applied for 2 h at room temperature or at 4 °C overnight. Subsequently, the samples were treated with the corresponding secondary antibody labeled with a fluorochrome (for human Slamf9 donkey anti-guinea pig Alexa488 and for human CD68 donkey anti-mouse Cy3 (both Dianova, Hamburg, Germany). Images were taken with the Nikon Eclipse NI microscope with the Clara interline CCD camera (Andor, Belfast, UK) NIS-Elements Advanced software (Nikon, Düsseldorf, Germany).

### In situ hybridization

In situ hybridization was performed with the RNAscope® 2.5 HD Detection Kit (ACDbio, Newark, CA, USA) to visualize Slamf9 mRNA in formalin-fixed paraffin-embedded melanomas. Initially, paraffin embedded tissue samples were deparaffinized with xylene and 100% ethanol and the slides were air-dried. Then, a hydrogen-peroxide block and the antigen retrieval were performed by boiling in Target Retrieval Solution for 15 min and Protease-Treatment for 30 min. The Slamf9 probe (20ZZ probe: Hs-SLAMF9 targeting 138–1139 of NM_033438.3) was hybridized for 2 h at 40 °C and the signal was amplified and detected with RED-A and -B solution followed by counterstaining with 50% hematoxylin. Pictures were taken with a Leica DCRE microscope, Leica DC500 camera, and software system (Leica, Wetzlar, Germany).

### Scratch assay

An artificial wound was caused to a 100% confluent monolayer of RAW 264.7 cells with a 10 μl pipette tip. Floating cells were washed away and scratch areas were photographed every 2 h after the initial scratch with Axio Vert.A1 microscope (Zeiss, Jena, Germany). Five representative pictures were taken of each scratch at each time point. Of each picture, 5 distances between scratch borders were measured with ImageJ software. The area under the curve was calculated with GraphPad Prism 6.0 (GraphPad Software, USA) for all three independent experiments. Statistics were calculated by using Student’s *t*-test.

### Western blot

Cells were lysed with RIPA-P buffer. Proteins were separated by gel electrophoresis using 10% SDS-polyacrylamide gels. After semi-dry blotting (Bio-Rad) onto PVDF membranes (GE Healthcare), the blots were incubated with primary antibodies (rat anti-mmSlamf9 antibody, guinea pig anti-hsSLAMF9, anti-GAPDH antibody (Santa Cruz Biotechnology, Heidelberg, Germany) and respective isotype controls) overnight at 4 °C. Subsequently, blots were incubated with secondary antibodies (anti-guinea pig HRP or anti-rat IgG1 HRP (both Dianova, Hamburg, Germany) and for signal detection, SuperSignal West Pico Chemiluminescent Substrate (Thermo Fisher Scientific) was used. Signal detection was performed using c600 Western Blot Imaging System (Azure Biosystems, Dublin, CA, USA).

### Quantitative RT-PCR analysis

RNA was isolated using innuPREP RNA Mini Kit (Jena Analytik, Jena, Germany) according to the manufacturer’s protocol. For reverse transcription 1 μg of RNA per sample was used. The reaction was performed with Maxima Reverse Transcriptase using Oligo(dT)_18_ primer (both Thermo Fisher Scientific, Munich, Germany). Prior to qRT-PCR, template cDNA was diluted 1:40 in ddH_2_O, mixed with primers (2 μM), as well as SyBRGreen Master Mix (Applied Biosystems by Thermo Fisher Scientific, Munich, Germany) according to the manufacturer’s protocol. The PCR was performed under standard conditions in a MX3000P sequence detection system (Stratagene by Agilent, Waldbronn, Germany).

### Generation of transgenic cell lines

Human Slamf9 cDNA (clone IRATp970E11113D, SourceBioscience, Nottingham, UK) or murine Slamf9 cDNA (clone IRAVp968B0535D, SourceBioscience, Nottingham, UK) was amplified by PCR and cloned into a modified lentiviral expression system vector pHAGE, denominated ADR3 in the following^34^. HEK293/T cells were transfected with 3rd generation lentiviral plasmids (pMD2.G L1, pRSV rev L2, pMDLg/pRRE L3, and pCDNA3.1/p35 E 71) in combination with ADR3-SLAMF9 or the empty vector (EV) as a control. Positive selection was performed using puromycin (2 µg/ml, Thermo Fisher Scientific, Munich, Germany), after U937 and RAW 264.7 were infected with the produced lentiviruses.

### Enzyme-linked immunosorbent assay

Prior to the assay, cell culture supernatants were centrifuged to obtain cell-free culture media, the resulting supernatant was transferred to fresh tubes. Assays were performed according to the manufacturer’s instructions (Mouse and human TNF-α DuoSet ELISA, R&D Systems, Wiesbaden, Germany).

### cDNA microarray analysis

The gene expression analysis was performed with the mouse genome 430 2.0 DNA microarray (Affymetrix by Thermo Fisher Scientific, Munich, Germany) according to the manufacturer’s instructions. Differential gene expression was assessed with commercially available software SAS JMP7 Genomics 3.2 based on log-linear mixed-model Anova.

### Statistics

Statistical analysis of all data were calculated by using GraphPad Prism 6.0 (GraphPad Software, USA). Statistical significance was examined by using Student’s *t*-test or by one-way ANOVA and Bonferroni as a post test. The level of significance is indicated by asterisks (****≤0.0001; ***≤0.001; **≤0.01, and *≤0.05). Error bars depict standard error of mean (SEM) of each experiment. All experiments were performed at least in triplicates.

## Electronic supplementary material

Supplemental figures
