# Supplementary material for: The novel immunoglobulin super family receptor SLAMF9 identified in TAM of murine and human melanoma influences pro-inflammatory cytokine secretion and migration
Source: Cell Death Dis. 2018 Sep 19;9(10):939. doi: 10.1038/s41419-018-1011-1 (PMC6145869; doi:10.1038/s41419-018-1011-1)
Supplement: Supplementary file 1 — Supplemental figures [file 41419_2018_1011_MOESM1_ESM.docx]

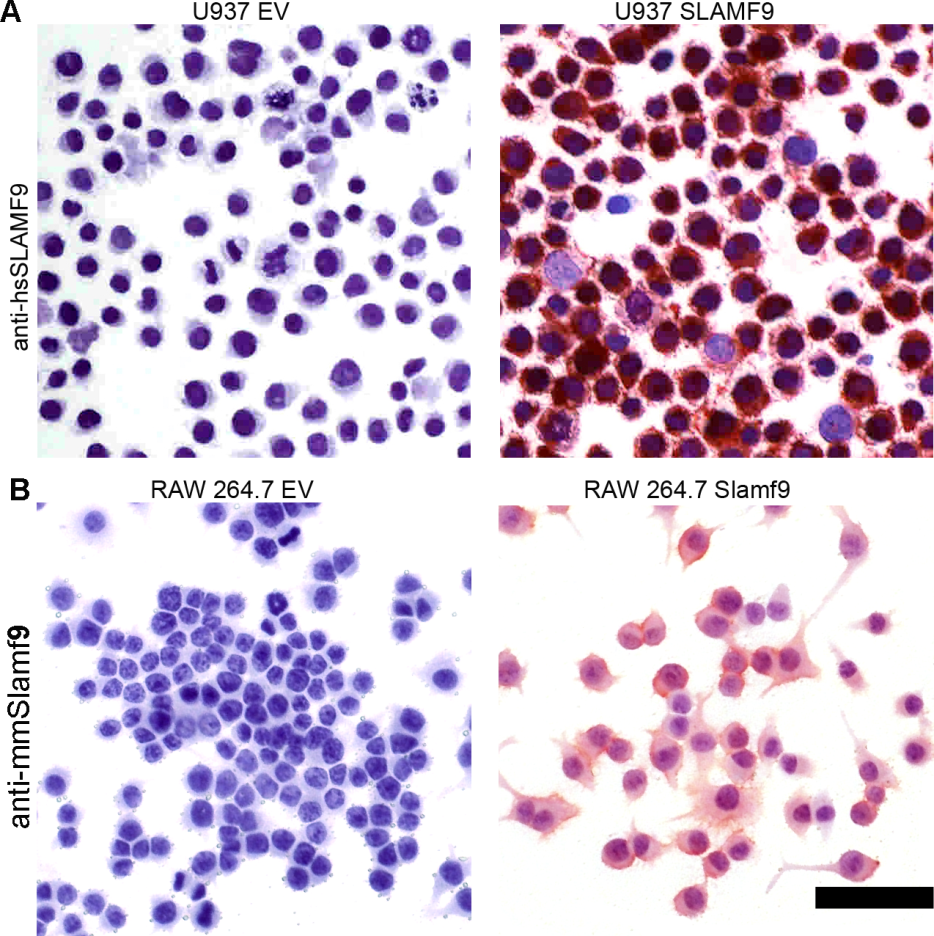


Supplemental Figure 1: Validation of anti-SLAMF9 antibody specificity by immunocytochemical staining of SLAMF9^+^ transgenic cell lines.

(A) Cytospins of human U937 EV and U937 SLAMF9^+^ cells were fixed with acetone and stained with self-generated polyclonal anti-hsSLAMF9, scale bars = 50 μm.

(B) Murine RAW 264.7 EV and RAW 264.7 Slamf9^+^ cells were grown on coverslips and acetone-fixed. Staining was performed with the generated monoclonal anti-mmSLAMF9 antibody.


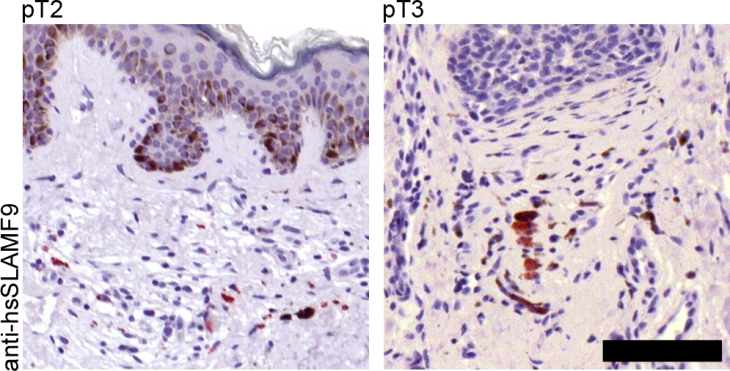


Supplemental Figure 2: Identification and quantification of SLAMF9^+^ cells in benign and malignant melanocytic naevi.

Human melanoma specimens were immunohistochemically stained with a self-generated polyclonal anti-hsSLAMF9 antibody. Representative pictures of pT2 and pT3 melanoma specimens are shown, scale bar = 100 µm.


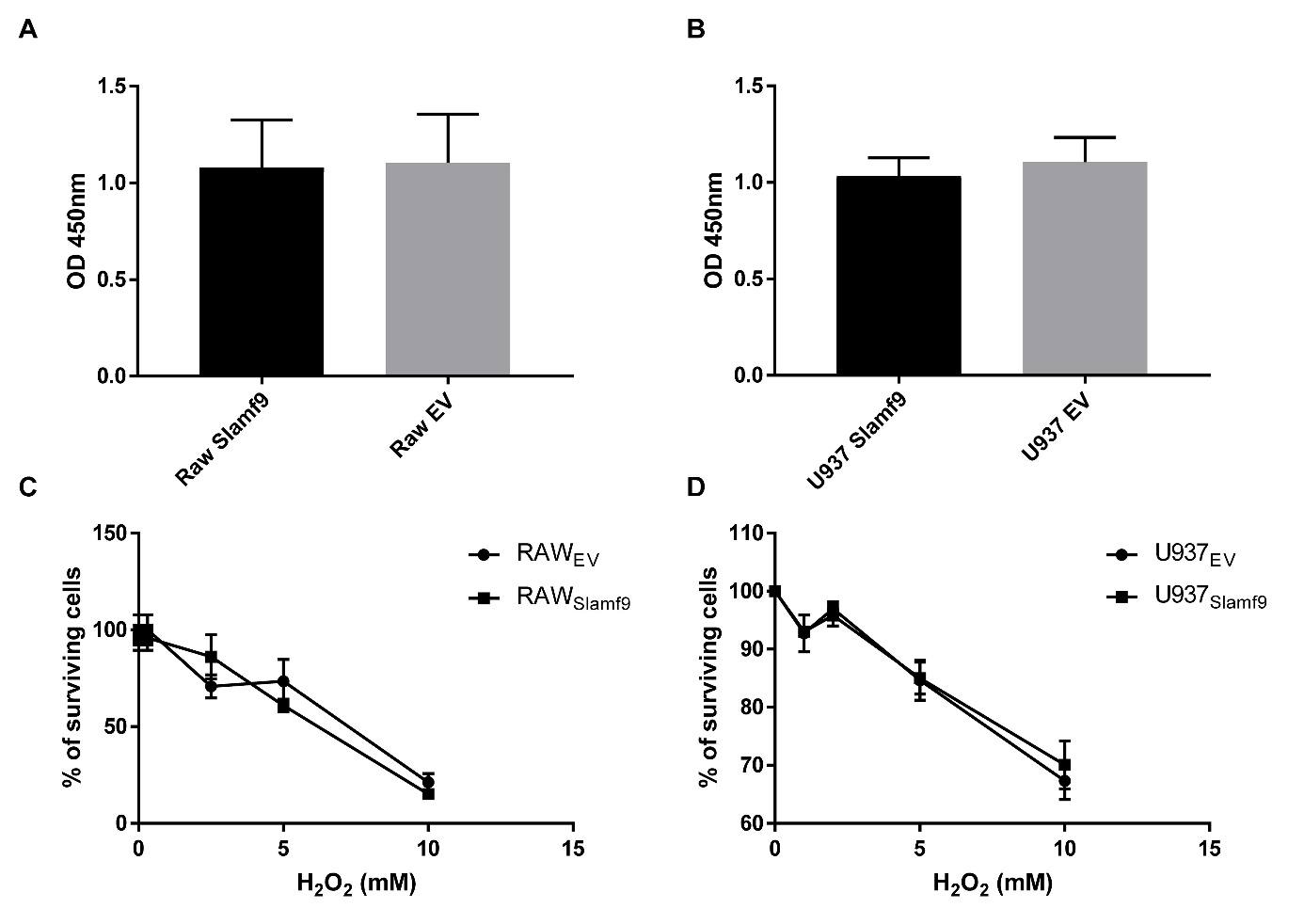


Supplemental Figure 3: Transgenic overexpression of SLAMF9 does not influence proliferation or H_2_O_2_ induced cell death of RAW 264.7 and U937 cells.

(A) SLAMF9^+^ Raw and U937 cells were pulsed with BrdU for 4h and labeled with an anti-BrdU monoclonal antibody (BrdU Cell proliferation ELISA Kit colorimetric, Abcam, Cambridge, UK). Optical density at 450nm was measured by using a microplate reader, n = 3.

(B) Transgenic RAW 264.7 and U937 cells were stimulated with various concentrations of H_2_O_2_ for 3 h. 7-AAD staining was performed and the number of 7-AAD negative cells (live cells) was assessed by FACS. The percentage of surviving cells is given in relation to untreated cells (0 mM H_2_O_2_). n = 3

Supplemental Table 1: Sequences of primers used for RT-PCR.

| Primer | Orientation | 5’−3’ Sequence | product length |
| --- | --- | --- | --- |
| mmSlamf1 | *for* | gcc cca gag tct gtc cag gaa cc | 262 |
|  | *rev* | tgc atg ccc agg aag cct cac |  |
| mmSlamf2 | *for* | agt att tga tcc tgt gcc caa gcc | 318 |
|  | *rev* | acc act agc caa gtt gca gtc ca |  |
| mmSlamf3 | *for* | gcc tcc cga gac ccc agt gt | 223 |
|  | *rev* | ccc agg aga cgt tga ggt gcg |  |
| mmSlamf4 | *for* | ggc caa gac tgc cca gat tct tc | 307 |
|  | *rev* | tat tgc aca ctt ttc cgc ctg tgt t |  |
| mmSlamf5 | *for* | tcc ttc aaa atc gtc cac tcc | 211 |
|  | *rev* | aac gga aca gaa atg cca ac |  |
| mmSlamf7 | *for* | gtc ggg cat ttc ctg gtg cct | 291 |
|  | *rev* | tgc cac cct ctc cat cgc ct |  |
| mmSlamf8 | *for* | atg gtg gat aca agg ggt ca | 293 |
|  | *rev* | atg cag gta aag gcc aca tc |  |
| mmSlamf9 | *for* | cct agc cag ctg acc aag tc | 369 |
|  | *rev* | gcg ttg taa agc cct gag tc |  |
| mmActin | *for* | tct acg agg gct atg ctc tcc | 330 |
|  | *rev* | gga tgc cac agg att cca tac |  |
| mmArg1 | *for* | aag ctg gtc tgc tgg aaa aa | 310 |
|  | *rev* | ctg gtt gtc agg gga gtg tt |  |
| mmHP | *for* | ggg agc tgt tgt cac tct cc | 249 |
|  | *rev* | tca cat tcg ggg agt ttc tc |  |
| mmLCN2 | *for* | cca gtt cgc cat ggt att tt | 206 |
|  | *rev* | cac act cac cac cca ttc ag |  |
| mmMMP9 | *for* | cgt cgt gat ccc cac tta ct | 225 |
|  | *rev* | aac aca cag ggt ttg cct tcc |  |
| mmSAA3 | *for* | tga cag cca aag atg ggt cca gt | 232 |
|  | *rev* | Gtc ctc tgc tcc atg tcc cgt ga |  |
| hsSLAMF9 | *for* | tgg tgt gct ctg tgg aga ag | 324 |
|  | *rev* | ttt tct gga ctc gga tga cc |  |
| hsACTIN | *for* | ggc acc aca cct tct aca atg a | 387 |
|  | *rev* | tct cct taa tgt cac gca cga t |  |
